# Ischiopagus tetrapus conjoined twins: 22 years after separation

**DOI:** 10.4103/0971-9261.54809

**Published:** 2009

**Authors:** Subir K. Chatterjee, Amal K. Chakravarty, N. Demajumdar, Kalyan K. Sarkar, Sukanta Misra

**Affiliations:** Department of Pediatric Surgery, Ramakrishna Mission Sevapratisthan and Vivekananda Institution of Medical Sciences, Kolkata, India; 1 Department of Urology, Ramakrishna Mission Sevapratisthan and Vivekananda Institution of Medical Sciences, Kolkata, India; 2 Department of Orthopedic Surgery (Rtd), Ramakrishna Mission Sevapratisthan and Vivekananda Institution of Medical Sciences, Kolkata, India; 3 Department of Obstetrics and Gynecology, Ramakrishna Mission Sevapratisthan and Vivekananda Institution of Medical Sciences, Kolkata, India

**Keywords:** Conjoined twins, ischiopagus twins, twins seperation

## Abstract

There is no record of both separated ischiopagus tetrapus conjoined twins leading normal lives 22 years after separation. We separated a pair of such twins in 1986 and have followed them up till date. Details regarding the technique of separation and the procedures required to ensure normal defecation and micturition and normal musculoskeletal function have been described by us in earlier communications. In this paper we describe their present status and ability to face the world as independent adult females. The outcome reflects the responsibilities and dedication of pediatric surgeons for infant patients with congenital problems after they have become adults, taking a pivotal role to involve surgeons of other disciplines as and when necessary.

## INTRODUCTION

There is no record of both separated ischiopagus tetrapus conjoined twins leading normal lives 22 years after separation. We separated a pair of such twins in 1986 and have followed them up till date. Details regarding the technique of separation and the procedures required to ensure normal defecation and micturition and normal musculoskeletal function have been described by us in earlier communications.[1–5] In this paper we describe their present status and their ability to face the world as independent adult females.

There are very few reports of successful separation of ischiopagus tetrapus conjoined twins prior to 1986. The ones that we had studied prior to undertaking the separation of our twins had emanated from Chapel Hill,[6] Munich[7] and Kuala Lumpur.[8] In 1989, Hoyle *et al*.[9] recorded the follow-up of the Chapel Hill twins. One died at the age of two years and the other, at 23 years, survived with gross musculoskeletal deformities.

## CASE REPORT

**Figure 1:**
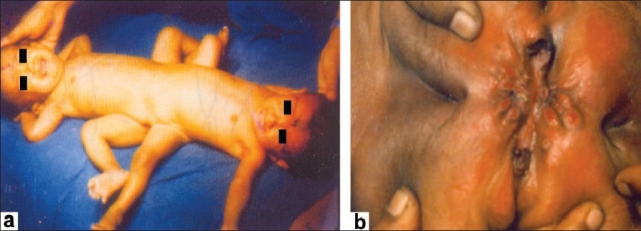
Photographs of the twins at first presentation; (a) and of their perineum (b) 1986

The twins, Ganga and Jamuna, came to us from a remote town in the North Eastern corner of our country in April, 1986 when they were five months old [Figures 1a and b]. They weighed eight kg. We assessed the extent of sharing by clinical examination and the two imaging parameters available to us at that time, conventional X-Ray and a primitive form of isotope scintigraphy.

The structures that they shared were intestinal, musculoskeletal, and urogenital.

### Intestinal

The only intestinal structure shared was the large bowel, beginning with a single cecum into which both ileums entered and ending in a single anus in the territory of Ganga. This large bowel was chronically obstructed and there were bouts of enterocolitis. The general condition of the twins was poor and their weight fell to seven kg. Hence, we decided to stage the separation. We carried out the first stage in August 1986. Both twins were left with end colostomies and the anus and anal canal remained with Ganga. After this operation the nutritional status of the twins improved greatly and we could ensure that fecal contamination would not occur during the definitive separation. The definitive separation performed in November 1986 was uneventful. Ganga was left with a perineum with three openings; urethral, vaginal and anal, Jamuna's perineum had only two openings; urethral and vaginal. The end colostomies were retained. The operative procedures were described in 1988.[1]

Intestinal reconstruction was carried out in 1989 and 1990 after the twins were rehabilitated in an SOS village in Kolkata. Subsequently they managed by bowel training till 1998. In that year we performed an anorectal myectomy on Ganga because we felt that her constipation was due to residual aganglionosis and we performed a revisional posterior sagittal anorectoplasty on Jamuna to deal with her soiling. Details have been described by us in 1998.[3] Both twins improved greatly after these procedures; they have remained clean and have been passing stool well since then with minimum laxative.

### Musculoskeletal

**Figure 2:**
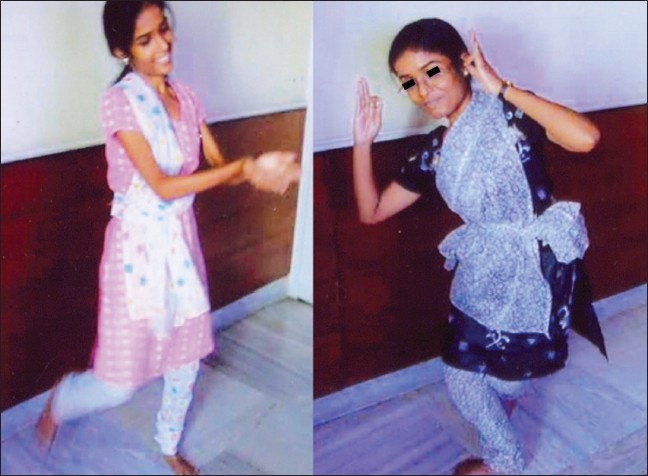
Photograph of the twins in 2008 in a dance sequence

The skeletal separation involved converting a large ‘O’ shaped pelvis into two smaller ‘O’ shaped pelvises; after this the lower limbs which were at right angles to the trunk came together in the line of the trunk. Details have been described earlier in 1991.[2] Sixteen months after separation, they learnt to stand, walk and even run. Today they engage in dancing and competitive sports [Figure 2]. However, X-Rays show some separation of Ganga's pubes and total disappearance of Jamuna's right pubis.

### Urogenital

The twins had four normal kidneys and ureters and two normal bladders and urethras. However, the bladders and urethras were retro pubic and each bladder received one ureter from each twin. During separation all the pelvic organs had to be rotated through 90 degree so that they could be placed within the trunk of each twin. The right ureter and all the vessels and nerves reaching the bladder and the genital organs from the right side were divided. After the rotation, Ganga's right ureter was reconstructed by an end to end ureteroureterostomy and a neoureterocystostomy (Cohen) was done on Jamuna. After the catheters were removed both twins micturated more or less normally.

Over the next few years both twins had a manageable amount of urinary soiling and a few episodes of urinary infection. They were managed conservatively till 2007; then after a full urological investigation they were submitted to major corrective surgery. They recovered uneventfully and now, both have normal continence and normal frequency. Details have been described elsewhere.[5] It is gratifying that there is near normal voiding function in our now adult patients clinically and urodynamicaly despite extensive surgery with division of one half of the vesical innervation.

Turning now to the genital organs, the twins were born with a pair of vulvas with urethral and vaginal orifices below the two pubic arches. During separation, bicornuate uteruses with two normal ovaries and tubes were found between the retro pubic urinary bladders and the single rectum. After the 90 degree rotation of the pelvic organs, each twin received a complete set of pelvic urogenital organs minus the blood vessels and nerves from the right side. The vagina allotted to Ganga was septate while Jamuna's vagina was single distally and septation started higher up.

In 1999, Jamuna had an episode of acute left lower abdominal pain; hematometros was diagnosed and drained and the stenotic opening of the cervix was dilated to ensure free flow of uterine contents into the vagina. There were no problems since and both twins have had normal periods. At present Jamuna has a capacious vagina admitting an index finger, but there is very little space between the vestibule and the neo anus. The two halves of Ganga's septate vagina admit bougies but there is no space at all between the vestibule and the native anus.

Successful pregnancy in a separated omphalopelvo ischiopagus tetrapus twin aged 20 years was reported in 1994.[10] We are therefore hopeful about the reproductive potential of both our twins although there is a high chance of miscarriage in all three trimesters.

## DISCUSSION

All the members of the teams which looked after the twins at various periods of their life have contributed to the result. In addition, the twins would certainly not have done as well as they have if they had not been looked after by the dedicated staff of the SOS Children's village where they have lived all these years. An organization like this is ideal for abandoned babies who require not only food, shelter, education and love but also medical and surgical care from time to time. The pediatric surgeons involved in the separation continued to follow up the twins even after they became adults and called consultants of other disciplines after ensuring that these consultants would give their time and expertise for a congenital problem already being handled by others

We have often pondered over the possibility of a better result if we had done things differently. In the state that the twins came, we could never have got away with a single operation. However, the staging could have been planned differently so that at the end of the separation both twins would be defecating per perineum Also, transuretero ureterostomies could have allowed us to reconstruct the urinary organs without having to use the partially devascularized terminal right ureters.

In conclusion, we entirely agree with ONeill when he wrote:

“No single discipline can expect to have all the necessary talents or completely up to date information on every conceivable reconstructive technique. Although it is critical to have a coordinating surgeon familiar with all aspects of surgical management, no single individual can or should do it all”.[11]

## References

[1] Chatterjee SK, Chakravarty AK, DebMaulik T, DeMajumdar N, Sen MK (1988). Staging the separation of ischiopagus twins. J Pediatr Surg.

[2] DeMajumdar N, Chatterjee SK, Chakraborty T, Chakravarty AK, Deb Maulik T, Sen MK (1991). Musculoskeletal problems in the separation of ischiopagus tetrapus twins. J Pediatr Ortho.

[3] Chatterjee SK, Chakravarty AK, Haque J (1999). Conjoined twins: Life after separation. Asian J Surg.

[4] Sarkar KK, Chatterjee SK, Chakravarty AK (2008). Urological status of ischiopagus tetrapus conjoined twins 22 years after separation: Paper presented at the annual conference of the Tamilnadu Pondicherry Chapter.

[5] DeMajumdar N (2009). Musculoskeletal status of ischiopagus tetrapus conjoined twins 22 years after separation: Paper presented at the annual conference of Pediatric Orthopedic Society of India.

[6] Eades JW, Thomas CG (1966). Successful separation of ischiopagus tetrapus conjoined twins. Ann Surg.

[7] Grantzow R, Hecker WC, Holschneider AM, Devens K, Helmig FJ (1984). Surgery on symmetrical conjoined ischiopagus twins. Z Kinderchir.

[8] Somasundaram K, Wong KS (1986). Ischiopagus tetrapus conjoined twins. Br J Surg.

[9] Hoyle RM, Thomas CG (1989). Twenty three year follow-up of separated ischiopagus tetrapus conjoined twins. Ann Surg.

[10] Shah LP, Chazotte C (1994). Successful pregnancy in a separated conjoined twin. Am J Obstet Gynecol.

[11] O'Neill JA, O'Neill, Rowe, Grosfeld, Fonkalsrud, Coran (1998). Conjoined twins – in pediatric surgery.

